# Fractional Norms and Quasinorms Do Not Help to Overcome the Curse of Dimensionality

**DOI:** 10.3390/e22101105

**Published:** 2020-09-30

**Authors:** Evgeny M. Mirkes, Jeza Allohibi, Alexander Gorban

**Affiliations:** 1 School of Mathematics and Actuarial Science, University of Leicester, Leicester LE1 7HR, UK; jhaa1@leicester.ac.uk (J.A.); a.n.gorban@leicester.ac.uk (A.G.); 2 Laboratory of Advanced Methods for High-Dimensional Data Analysis, Lobachevsky State University, 603105 Nizhny Novgorod, Russia; 3 Department of Mathematics, Taibah University, Janadah Bin Umayyah Road, Tayba, Medina 42353, Saudi Arabia

**Keywords:** curse of dimensionality, blessing of dimensionality, kNN, metrics, high dimension, fractional norm

## Abstract

The curse of dimensionality causes the well-known and widely discussed problems for machine learning methods. There is a hypothesis that using the Manhattan distance and even fractional $l_{p}$ quasinorms (for *p* less than 1) can help to overcome the curse of dimensionality in classification problems. In this study, we systematically test this hypothesis. It is illustrated that fractional quasinorms have a greater relative contrast and coefficient of variation than the Euclidean norm $l_{2}$, but it is shown that this difference decays with increasing space dimension. It has been demonstrated that the concentration of distances shows qualitatively the same behaviour for all tested norms and quasinorms. It is shown that a greater relative contrast does not mean a better classification quality. It was revealed that for different databases the best (worst) performance was achieved under different norms (quasinorms). A systematic comparison shows that the difference in the performance of kNN classifiers for $l_{p}$ at *p* = 0.5, 1, and 2 is statistically insignificant. Analysis of curse and blessing of dimensionality requires careful definition of data dimensionality that rarely coincides with the number of attributes. We systematically examined several intrinsic dimensions of the data.

## 1. Introduction

The term “curse of dimensionality” was introduced by Bellman [1] in 1957. Nowadays, this is a general term for problems related to high dimensional data, for example, for Bayesian modelling [2], nearest neighbour prediction [3] and search [4], neural networks [5,6], radial basis function networks [7,8,9,10] and many others. Many authors have studied the “meaningfulness” of distance based classification [11,12,13], clustering [12,14] and outlier detection [13,15] in high dimensions. These studies are related to the concentration of distances, which means that in high dimensional space the distances between almost all pairs of points of a random finite set have almost the same value (with high probability and for a wide class of distributions).

The term “blessing of dimensionality” was introduced by Kainen in 1997 [16]. The “blessing of dimensionality” considers the same effect of concentration of distances from the different point of view [17,18,19]. The concentration of distances was discovered in the foundation of statistical physics and analysed further in the context of probability theory [20,21], functional analysis [22], and geometry (reviewed by [23,24,25]). The blessing of dimensionality allows us to use some specific high dimensional properties to solve problems [26,27]. One such property is the linear separability of random points from finite sets in high dimensions [24,28]. A review of probability in high dimension, concentration of norm, and many other related phenomena is presented in [20].

The curse of dimensionality was firstly described 59 years ago [1] and the blessing of dimensionality was revealed 23 years ago [16]. The importance of both these phenomena increases in time. The big data revolution leads to an increase of the data dimensionality, and classical machine learning theory becomes useless in the post-classical world where the data dimensionality often exceeds the sample size (and it usually exceeds the logarithm of the sample size that makes many classical estimates pointless) [26]. The curse and blessing of dimensionality are two sides of the same coin. A curse can turn into a blessing and vice versa. For example, the recently found phenomenon of stochastic separability in high dimensions [24,29] can be considered as a “blessing” [28] because it is useful for fast non-iterative corrections of artificial intelligence systems. On the other hand, it can be considered as a “curse” [30]: the possibility to create simple and efficient correctors opens, at the same time, a vulnerability and provides tools for stealth attacks on the systems.

Since the “curse of dimensionality” and the “blessing of dimensionality” are related to the concept of high dimensionality, six different approaches to evaluation of dimension of data were taken into consideration. Beyond the usual dimension of vector space [31] (the number of atteributes), we considered three dimensions determined by linear approximation of data by principal components [32,33] with the choice of the number of principal components in accordance with the Kaiser rule [34,35], the broken stick rule [36] and the condition number of the covariance matrix [28,37]. We also considered the recently developed separability dimension [28,38] and the fractal dimension [39]. We demonstrated on many popular benchmarks that intrinsic dimensions of data are usually far from the dimension of vector space. Therefore, it is necessary to evaluate the intrinsic dimension of the data before considering any problem as high- or low-dimensional.

The $l_{p}$ functional ${∥x∥}_{p}$ in a *d* dimensional vector space is defined as

(1) $${∥x∥}_{p}={\left(\sum_{i=1}^{d}{\left|x_{i}\right|}^{p}\right)}^{1/p}.$$

The Euclidean distance is $l_{2}$ and the Manhattan distance is $l_{1}$. It is the norm for p≥1 and the quasinorm for 0<p<1 due to violation of the triangle inequality [40]. We consider only the case with p>0. It is well known that for p<q we have ${∥x∥}_{p}\geq {∥x∥}_{q},\forall x$.

Measuring of dissimilarity and errors using subquadratic functionals reduces the influence of outliers and can help to construct more robust data analysis methods [14,41,42]. The use of these functionals for struggling with the curse of dimensionality was proposed in several works [14,42,43,44,45,46]. In particular, Aggarwal et al. [14] suggested that “fractional distance metrics can significantly improve the effectiveness of standard clustering algorithms”. Francois, Wertz, and Verleysen studied Relative Contrast (RC) and Coefficient of Variation (CV) (called by them ‘relative variance’) of distances between datapoints in different $l_{p}$ norms [42]. They found that “the ‘optimal’ value of *p* is highly application dependent”. For different examples, the optimal *p* was equal to 1, 1/2, or 1/8 [42]. Dik et al. [43] found that for fuzzy c-means usage of $l_{p}$-quasinorms with 0<p<0.5 “improves results when compared to p≥0.5 or the usual distances, especially when there are outliers.” The purity of clusters was used for comparison. Jayaram and Klawonn [44] studied RC and CV for quasinorms without triangle inequality and for metrics unbounded on the unite cube. In particular, they found that indicators of concentration of the norm are better for lower *p* and, moreover, that unbounded distance functions whose expectations do not exist behave better than norms or quasinorms. France [45] compared effectiveness of several different norms for clustering. They found that the normalised metrics proposed in [46] give a better results and recommended to use the normalised $l_{1}$ metrics for nearest neighbours recovery.

In 2001, C.C. Aggarwal and co-authors [14] briefly described the effect of using fractional quasinorms for high-dimensional problems. They demonstrated that using of $l_{p}$ (p≤1) can compensate the concentration of distances. This idea was used further in many works [13,47,48]. One of the main problems of using the quasinorm $l_{p}$ for p<1 is time of calculation of minimal distances and solution of optimization problems with $l_{p}$ functional (which is even non-convex for p<1). Several methods have been developed to speed up the calculations [47,49].

The main recommendation of [14] was the use of Manhattan distance instead of Euclidean one [50,51,52]. The main reason for this is that a smaller *p* is expected to give better results but for p<1 the functional $l_{p}$ is not a norm, but a non-convex quasinorm. All methods and algorithms that assume triangle inequality [51,53,54] cannot use such a quasinorm.

A comparison of different $l_{p}$ functionals for data mining problems is needed. In light of the published preliminary results, for example, [14,55,56], more testing is necessary to evaluate the performance of data mining algorithms based on these $l_{p}$ norms and quasinorms.

In our study, we perform systematic testing. In general, we demonstrated that the concentration of distances for $l_{p}$ functionals was less for smaller *p*. Nevertheless, for all *p*, the dependences of distance concentration indicators (RC${}_{p}$ and CV${}_{p}$) on dimension are qualitatively the same. Moreover, the difference in distance concentration indicators for different *p* decreases with increasing dimension, both for RC and CV.

The poor performance of k Nearest Neighbour (kNN) classifiers in high dimensions is used as a standard example of the “curse of dimensionality” for a long time, from the early work [11] to the deep modern analysis [57]. The kNN classifiers are very sensitive to used distance (or proximity) functions and, therefore, they are of particular interest to our research.

We have systematically tested the hypothesis that measuring of dissimilarity by subquadratic norms $l_{p}$(1≤p<2) or even quasinorms (0<p<1) can help to overcome the curse of dimensionality in classification problems. We have shown that these norms and quasinorms do not systematically and significantly improve performance of kNN classifiers in high dimensions.

In addition to the main result, some simple technical findings will be demonstrated below that can be useful when analyzing multivariate data. Two of them are related to the estimation of the dimension of the data, and the other two consider the links between the use of different $l_{p}$ norms, the concentration of distances and the accuracy of the kNN classifiers:

- The number of attributes for most of real life databases is far from any reasonable intrinsic dimensionality of data;
- The popular estimations of intrinsic dimensionality based on principal components (Kaiser rule and broken stick rule) are very sensitive to irrelevant attributes, while the estimations based on the condition number of the reduced covariance matrix is much more stable as well as the definitions based on separability properties or fractal dimension;
- Usage of $l_{p}$ functionals with small *p* does not prevent the concentration of distances;
- A lower value of a distance concentration indicator does not mean better accuracy of the kNN classification.

Our paper is organized as follows. In Section 2, we present results of an empirical test of distance concentration for relative contrast and coefficient of variation also known as relative variance. Section 3 introduces the six used intrinsic dimensions. In Section 4, we describe the approaches used for $l_{p}$ functionals comparison, the used databases and the classification quality indicators. In Section 5, six intrinsic dimensions are compared for the benchmark datasets. In Section 6, we compare performance of classifiers for different $l_{p}$ functionals. The ‘Discussion’ section provides discussion and outlook. The ‘Conclusion’ section presents conclusions.

All software and databases used in this study are freely available online [58]. Some results of this work were presented partially at IJCNN’2019 [59]: comparison of RC and CV (Section 2) and comparison of $l_{p}$ functionals for 11NN classifier (part of Section 6).

## 2. Measure Concentration

Consider a database *X* with *n* data points $X=\{x_{1},\ldots ,x_{n}\}$ and *d* real-valued attributes, $x_{i}=\left(x_{i1},\ldots ,x_{id}\right)$. *x* without index is the query point: the point for which all distances were calculated. We used for testing two types of databases: randomly generated databases with i.i.d. components from the uniform distribution on the interval [0,1] (this section) and real life databases (Section 4). The $l_{p}$ functional for vector *x* is defined by (1). For comparability of results with [14], in this study, we consider the set of norms and quasinorms used in [14] with one more quasinorm ($l_{0.01}$): $l_{0.01},l_{0.1},l_{0.5},l_{1},l_{2},l_{4},l_{10},l_{\infty }$.

**Figure 1:**
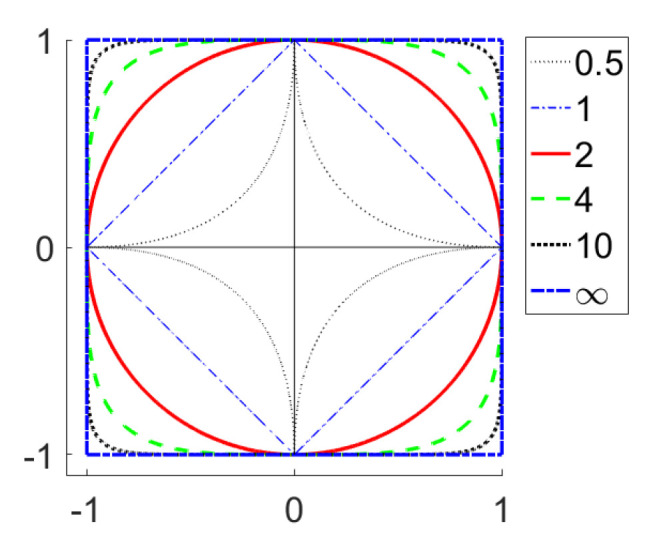
Unit level sets for $l_{p}$ functionals (“Unit spheres”).

Figure 1 shows the shapes of the unit level sets for all considered norms and quasinorms excluding $l_{0.01}$ and $l_{0.1}$. For two excluded quasinorms, the level sets are visually indistinguishable from the central cross.

Several different indicators were used to study the concentration of distances:

- Relative Contrast (RC) [11,14,42]
  (2) $${\mathrm{RC}}_{p}\left(X,x\right)=\frac{|\max_{i}∥x_{i}{-x∥}_{p}-\min_{i}∥x_{i}-x{∥}_{p}|}{\min_{i}{∥x_{i}-x∥}_{p}};$$
- Coefficient of Variations (CV) or relative variance [42,53,54]
  (3) $${\mathrm{CV}}_{p}\left(X,x\right)=\frac{\sqrt{var(∥x_{i}-x{∥}_{p}|)}}{mean(∥x_{i}-x{∥}_{p})},$$
  where var(z) is the variance and mean(z) is the mean value of the random variable *z*;
- Hubness (popular nearest neighbours) [13].

In our study, we used RC and CV. Hubness [13] characterised distribution of the number of *k*-occurrences of data points that is, the number of times the data point occurs among the k nearest neighbours of all other data points. With dimensionality increase, the distribution this *k*-occurrence becomes more skewed to the right, that indicates the emergence of hubs, i.e., popular nearest neighbours which appear in many more kNN lists than other points. We did not use hubness in our analysis because this change in the distribution of a special random variable, *k*-occurrence, needs additional convention about interpretation. Comparison of distributions is not so illustrative as comparison of real numbers.

**Figure 2:**
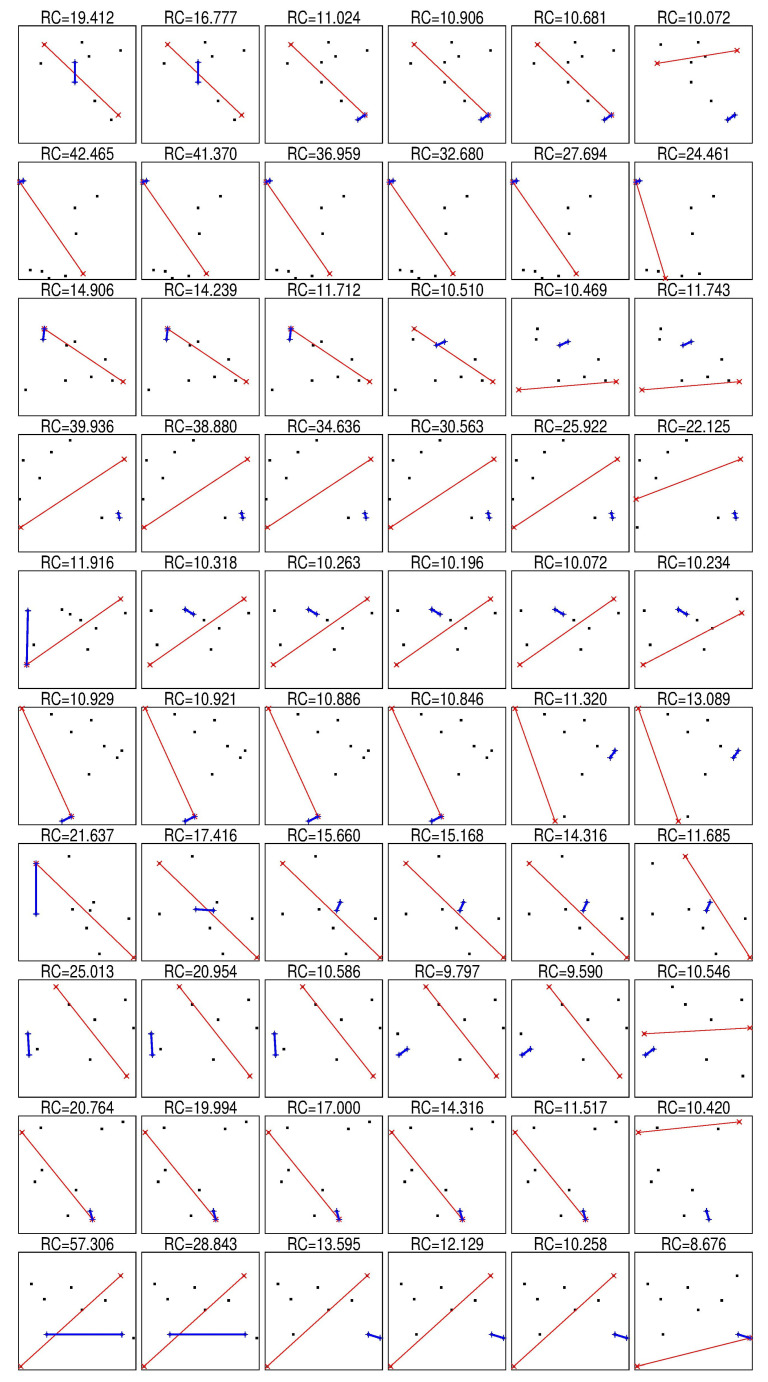
Ten randomly generated sets of 10 points, thin red line connects the furthest points and bold blue line connects closest points, columns (from left to right) corresponds to p=0.01,0.1,0.5,1,2,∞.

Table 2 in paper [14] shows that the proportion of cases where ${\mathrm{RC}}_{1}>{\mathrm{RC}}_{2}$ increases with dimension. It can be easily shown that for special choice of *X* and *x*, all three relations between RC${}_{1}$ and RC${}_{2}$ are possible: ${\mathrm{RC}}_{1}\left(X,x\right)>{\mathrm{RC}}_{2}\left(X,x\right)$ (all lines in Figure 2, exclude row 6), ${\mathrm{RC}}_{1}\left(X,x\right)={\mathrm{RC}}_{2}\left(X,x\right)$, or ${\mathrm{RC}}_{1}\left(X,x\right)<{\mathrm{RC}}_{2}\left(X,x\right)$ (row 6 in Figure 2). To evaluate the probabilities of these three outcomes, we performed the following experiment. We generated *X* dataset with *k* points and 100 coordinates. Each coordinate of each point was uniformly randomly generated in the interval [0,1]. For each dimension d=1,2,3,4,10,15,20,100, we created a *d*-dimensional dataset $X_{d}$ by selecting the first *d* coordinates of points in *X*. We calculated ${\mathrm{RC}}_{p}$ as the mean value of RC for each point in $X_{d}$:

(4) $${\mathrm{RC}}_{p}=\frac{1}{k}\sum_{i=1}^{k}{\mathrm{RC}}_{p}\left(X_{d}\backslash \left\{x_{i}\right\},x_{i}\right),$$

where X\{x} is the *X* database without the point *x*. We repeated this procedure 1000 times and calculated the fraction of cases when ${\mathrm{RC}}_{1}>{\mathrm{RC}}_{2}$. The results of this experiment are presented in Table 1. Table 1 shows that for k=10 points our results are very similar to the results presented in Table 2 in [14]. Increasing the number of points shows that even with a relatively small number of points (k≈20) for almost all databases ${\mathrm{RC}}_{1}>{\mathrm{RC}}_{2}$.

We can see that appearance of a noticeable proportion of cases when ${\mathrm{RC}}_{2}>{\mathrm{RC}}_{1}$ is caused by a small sample size. For not so small samples, in most cases ${\mathrm{RC}}_{2}<{\mathrm{RC}}_{1}$. This is mainly because the pairs of nearest (farthest) points can be different for different metrics. Several examples of such sets are presented in Figure 2. Figure 2 shows that ${\mathrm{RC}}_{2}<{\mathrm{RC}}_{\infty }$ in rows 3, 5, 6, and 8 and ${\mathrm{RC}}_{1}<{\mathrm{RC}}_{2}$ in row 6. These results allow us to formulate the hypothesis that in general almost always ${\mathrm{RC}}_{p}<{\mathrm{RC}}_{q},\forall p>q$. RC is widely used to study the properties of a finite set of points, but CV is more appropriate for point distributions. We hypothesise that ${\mathrm{CV}}_{p}<{\mathrm{CV}}_{q},\forall p>q$.

To check these hypotheses, we performed the following experiment. We created a *X* database with 10,000 points in 200 dimensional space. Each coordinate of each point was uniformly randomly generated in the interval [0,1]. We chose the set of dimensions d=1,2,3,4,5,10,15,…,195,200 and the set of $l_{p}$ functionals $l_{0.01},l_{0.1},l_{0.5},l_{1},l_{2},l_{4},l_{10},l_{\infty }$. For each dimension *d*, we prepared the $X_{d}$ database as the set of the first *d* coordinates of points in *X* database. For each $X_{d}$ database and $l_{p}$ functional, we calculate the set of all pairwise distances $D_{dp}$. Then, we estimated the following values:

(5) $${\mathrm{RC}}_{p}=\frac{\max D_{dp}-\min D_{dp}}{\min D_{dp}},{\mathrm{CV}}_{p}=\frac{\sqrt{var(D_{dp})}}{mean(D_{dp})}.$$

**Figure 3:**
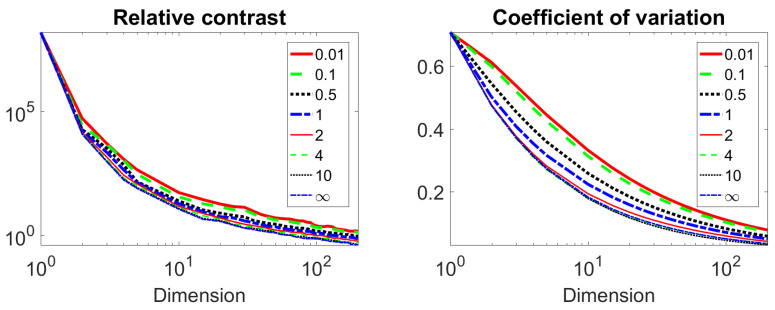
Changes of RC (**left**) and CV (**right**) with dimension for several metrics.

The graphs ${\mathrm{RC}}_{p}$ and ${\mathrm{CV}}_{p}$ are presented in Figure 3. Figure 3 shows that our hypotheses hold. We see that RC and CV as functions of dimension have qualitatively the same shape but in different scales: RC in the logarithmic scale. The paper [14] states that the qualitatively different behaviour of $\max_{i}∥x_{i}{∥}_{p}-\max_{i}{∥x_{i}∥}_{p}$ was observed for different *p*. We found qualitatively the same behavior for relative values (RC). The small quantitative difference ${\mathrm{RC}}_{p}-{\mathrm{RC}}_{q}$ increases for *d* from 1 to about 10 and decreases with a further increase in dimension. This means that there could be some preference for using lower values of *p* but the fractional metrics do not provide a panacea for the curse of dimensionality. To analyse this hypothesis, we study the real live benchmarks in the Section 4.

## 3. Dimension Estimation

To consider high dimensional data and the curse or blessing of dimensionality, it is necessary to determine what dimensionality is. There are many different notions of data dimension. Evaluation of dimensionality become very important with emergence of many “big data” databases. The number of attributes is the dimension of the vector space [31] (hereinafter referred to as #Attr). For the data mining tasks, the dimension of space is not as important as the data dimension and the intrinsic data dimension is usually less than the dimension of space.

The concept of intrinsic, internal or effective data dimensionality is not well defined for the obvious reason: the data sets are finite and, therefore, the direct application of topological definitions of dimension gives zero. The most popular approach to determining the data dimensionality is approximation of data sets by a continuous topological object. Perhaps, the first and at the same time widely used definition of intrinsic dimension is the dimension of the linear manifold of “the best fit to data” with sufficiently small deviations [32]. The simplest way to evaluate such dimension is Principal Component Analysis (PCA) [32,33]. There is no single (unambiguous) method for determining the number of informative (important, relevant, etc.) principal components [36,60,61]. The two widely used methods are the Kaiser rule [34,35] (hereinafter referred to as PCA-K) and the broken stick rule [36] (hereinafter referred to as PCA-BS).

Let us consider a *X* database with *n* data points $X=x_{1},\ldots ,x_{n}$ and *d* real-valued attributes, $x_{i}=\left(x_{i1},\ldots ,x_{id}\right)$. The empirical covariance matrix Σ(X) is symmetric and non-negative definite. The eigenvalues of the Σ(X) matrix are non-negative real numbers. Denote these values as $\lambda_{1}\geq \lambda_{2}\geq \cdots \geq \lambda_{d}$. Principal components are defined using the eigenvectors of empirical covariance matrix Σ(X). If the *i*th eigenvector $w_{i}$ is defined then the *i*th principal coordinate of the datavector *x* is the inner product $\left(x,w_{i}\right)$. The Fraction of Variance Explained (FVE) by *i*th principal component for the dataset *X* is

$$f_{i}=\frac{\lambda_{i}}{\sum_{j=1}^{d}\lambda_{j}}.$$

The Kaiser rule states that all principal components with FVE greater or equal to the average FVE are informative. The average FVE is 1/d. Thus, the components with $f_{i}\geq 1/d$ are considered as informative ones and should be retained and the components with $f_{i}<1/d$ should not. Another popular version uses a twice lower threshold 0.5/d and retains more components.

The broken stick rule compares the set $f_{i}$ with the distribution of random intervals that appear if we break the stick at d−1 points randomly and independently sampled from the uniform distribution. Consider a unit interval (stick) randomly broken into *d* fragments. Let us numerate these fragments in descending order of their length: $s_{1}\geq s_{2}\geq \cdots \geq s_{d}$. Expected length of *i* fragment is [36]

(6) $$b_{i}=\frac{1}{d}\sum_{j=i}^{d}\frac{1}{j}.$$

The broken stick rule states that the first *k* principal components are informative, where *k* is the maximum number such that $f_{i}\geq b_{i},\forall i\leq k$.

In many problems, the empirical covariance matrix degenerates or almost degenerates, that means that the smallest eigenvalues are much smaller than the largest ones. Consider the projection of data on the first *k* principal components: $\hat{X}=XV$, where the columns of the matrix *V* are the first *k* eigenvectors of the matrix Σ(X). Eigenvalues of the empirical covariance matrix of the reduced data $\Sigma (\hat{X})$ are $\lambda_{1},\lambda_{2},\ldots ,\lambda_{k}$. After the dimensionality reduction, the condition number (the ratio of the lowest eigenvalue to the greatest) [62] of the reduced covariance matrix should not be too high in order to avoid the multicollinearity problem. The relevant definition [28] of the intrinsic dimensionality refers directly to the condition number of the matrix $\Sigma (\hat{X})$: *k* is the number of informative principal components if it is the smallest number such that

(7) $$\frac{\lambda_{k+1}}{\lambda_{1}}<\frac{1}{C},$$

where *C* is specified condition number, for example, C=10. This approach is further referred to as PCA-CN. The PCA-CN intrinsic dimensionality is defined as the number of eigenvalues of the covariance matrix exceeding a fixed percent of its largest eigenvalue [37].

The development of the idea of data approximation led to the appearance of principal manifolds [63] and more sophisticated approximators, such as principal graphs and complexes [64,65]. These approaches provide tools for evaluating the intrinsic dimensionality of data and measuring the data complexity [66]. Another approach uses complexes with vertices in data points: just connect the points with distance less than ε for different ε and get an object of combinatorial topology, simplicial complex [67]. All these methods use an object embedded in the data space. They are called Injective Methods [68]. In addition, a family of Projective Methods was developed. These methods do not construct a data approximator, but project the dataspace onto a space of lower dimension with preservation of similarity or dissimilarity of objects. A brief review of modern injective and projective methods can be found in [68].

Recent studies of curse/blessing dimensionality introduce a new method for evaluation intrinsic dimension: separability analysis. A detailed description of this method can be found in [28,38] (hereinafter referred to as SepD). For this study, we used an implementation of separability analysis from [69]. The main concept of this approach is the α Fisher separability: point *x* of dataset *X* is α Fisher separable from dataset *X* if

(8) (x,y)≤α(x,x),∀y∈X,y≠x,

where (x,y) is dot product of vectors *x* and *y*.

The last intrinsic dimension used in this study is the fractal dimension (hereinafter referred to as FracD). It is also known as box-counting dimension or Minkowsk–Bouligand dimension. There are many versions of box-counting algorithms and we used R implementation from the RDimtools package [70]. The definition of FracD is

$$d_{f}=\lim_{r\to 0}\frac{\log(N(r))}{\log(1/r)},$$

where *r* is the size of the *d*-cubic box in the regular grid and N(r) is the number of cells with data points in this grid. Of course, formally this definition is controversial since the data set is finite and there is no infinite sequence of discrete sets. In practice, the limit is substituted by the slope of the linear regression for sufficiently small *r* but without intercept.

There are many approaches to non-linear evaluation of data dimensionality with various non-linear data approximants: manifolds, graphs or cell complexes [63,65,66,68]. The technology of neural network autoencoders is also efficient and very popular but its theoretical background is still under discussion [71]. We did not include any other non-linear dimensionality reduction methods in our study because there is a fundamental uncertainty: it is not known a priori when to stop the reduction. Even for simple linear PCA, we have to consider and compare three stopping criteria, from Kaiser rule to the condition number restriction. For non-linear model reduction algorithms the choice of possible estimates and stopping criteria is much richer. The non-linear estimates of the dimensionality of data may be much smaller than the linear ones. Nevertheless, for the real life biomedical datasets, the difference between linear and non-linear dimensions is often not so large (from 1 to 4), as it was demonstrated in [65].

## 4. Comparison of *l_p_* Functionals

In Section 2, we demonstrated that ${\mathrm{RC}}_{p}$ is greater for smaller *p*. It was shown in [11] that greater RC means ‘more meaningful’ task for kNN. We decided to compare different $l_{p}$ functions for kNN classification. Classification has one additional advantage over regression and clustering problems: the standard criteria of classification quality are classifier independent and and do not depend on the dissimilarity measures used [72].

For this study, we selected three classification quality criteria: the Total Number of Neighbours of the Same Class (TNNSC) (that is, the total number of the *k* nearest neighbors that belonged to the same class as the target object over all the different target objects), accuracy (fraction of correctly recognised cases), sum of sensitivity (fraction of correctly solved cases of positive class) and specificity (fraction of correctly solved cases of negative class). TNNSC is not an obvious indicator of classification quality and we use it for comparability of our results with [14]. kNN with 11 nearest neighbours was used also for comparability with [14].

### 4.1. Databases for Comparison

We selected 25 databases from UCI Data repository [73]. To select the databases, we applied the following criteria:

1. Data are not time-series.
2. Database is formed for the binary classification problem.
3. Database does not contain any missing values.
4. The number of attributes is less than the number of observations and is greater than 3.
5. All predictors are binary or numeric.

In total, 25 databases and 37 binary classification problems were selected (some databases contain more than one classification problem). For simplicity, we refer to each task as a ‘database’. The list of selected databases is presented in Table 2.

We do not set out to determine the best database preprocessing for each database. We just use three preprocessing for each database:

- Empty preprocessing means usage data ‘as is’;
- Standardisation means shifting and scaling data to have a zero mean and unit variance;
- Min-max normalization refers to shifting and scaling data in the interval [0,1].

### 4.2. Approaches to Comparison

Our purpose is to compare $l_{p}$ functionals but not to create the best classifier for each problem. Following [14], we use the 11NN classifier, and 3NN, 5NN and 7NN classifiers for more general result. One of the reasons for choosing kNN is strong dependence of kNN on used metrics and, on the other hand, the absence of any assumption about the data, excluding the principle: tell me your neighbours, and I will tell you what you are. In our study, we consider kNN with $l_{0.01},l_{0.1},l_{0.5},l_{1},l_{2},l_{4},l_{10},l_{\infty }$ as different algorithms. We applied the following indicators to compare kNN classifiers (algorithms) for listed $l_{p}$ functionals:

- The number of databases for which the algorithm is the best [107];
- The number of databases for which the algorithm is the worst [107];
- The number of databases for which the algorithm has performance that is statistically insignificantly different from the best;
- The number of databases for which the algorithm has performance that is statistically insignificantly different from the worst;
- The Friedman test [108,109] and post hoc Nemenyi test [110] which were specially developed to compare multiple algorithms;
- The Wilcoxon signed rank test was used to compare three pairs of metrics.

We call the first four approaches frequency comparison. To avoid discrepancies, a description of all used statistical tests is presented below.

#### 4.2.1. Proportion Estimation

Since two criteria of classification quality – accuracy and TNNSC/(k×n), where *n* is the number of cases in the database – are proportions, we can apply *z*-test for proportion estimations [111]. We want to compare two proportions with the same sample size, so we can use a simplified formula for test statistics:

(9) $$z=\frac{|p_{1}-p_{2}|}{\sqrt{\frac{p_{1}+p_{2}}{n}\left(1-\frac{p_{1}+p_{2}}{2}\right)}},$$

where $p_{1}$ and $p_{2}$ are two proportions for comparison. *p*-value of this test is the probability of observing by chance the same or greater *z* if both samples are taken from the same population. *p*-value is $p_{z}=\Phi \left(-z\right)$, where Φ(z) is the standard cumulative normal distribution. There is a problem of reasonable choice of significance level. The selected databases contain from 90 to 130,064 cases. Using the same threshold for all databases is meaningless [112,113]. The required sample size *n* can be estimated through the specified significance level of 1−α, the statistical power 1−β, the expected effect size *e*, and the population variance $s^{2}$. For the normal distribution (since we use *z*-test) this estimation is:

(10) $$n=\frac{2{\left(z_{1-\alpha }+z_{1-\beta }\right)}^{2}s^{2}}{e^{2}}.$$

In this study, we assume that the significance level is equal to the statistical power α=β, the expected effect size is 1% (1% difference in accuracy is large enough), and the population variance can be estimated by the formula

(11) $$s^{2}=n\frac{n_{+}}{n}\left(1-\frac{n_{+}}{n}\right)=\frac{n_{+}\left(n-n_{+}\right)}{n},$$

where $n_{+}$ is the number of cases in the positive class. Based on this assumption, we can estimate a reasonable level of significance as

(12) $$\alpha =\Phi \left(\frac{e}{s}\sqrt{\frac{n}{8}}\right).$$

Usage of eight $l_{p}$ functionals means multiple testing. To avoid overdetection problem we apply Bonferroni correction [114]. On the other hand, usage of too high a significance level is also meaningless [112]. As a result, we select the significance level as

(13) $$\alpha =\max\left\{\frac{1}{28}\Phi \left(\frac{e}{s}\sqrt{\frac{n}{8}}\right),0.00001\right\}.$$

The difference between two proportions (TNNSC or accuracy) is statistically significant if $p_{z}<\alpha$. It must be emphasized that for TNNSC the number of cases is kn because we consider *k* neighbours for each point.

#### 4.2.2. Friedman Test and Post Hoc Nemenyi Test

One of the widely used statistical tests for comparing algorithms on many databases is the Friedman test [108,109]. To apply this test, we firstly need to apply the tied ranking for the classification quality score for one database: if several classifiers provide exactly the same quality score then the rank of all such classifiers will be equal to the average value of the ranks for which they were tied [109]. We denote the number of used databases as *N*, the number of used classifiers as *m* and the rank of classifier *i* for database *j* as $r_{ji}$. The mean rank of classifier *i* is

(14) $$R_{i}=\frac{1}{N}\sum_{j=1}^{N}r_{ji}.$$

Test statistics is

(15) $$\chi_{F}^{2}=\frac{N^{2}\left(m-1\right)\left(4\sum_{i=1}^{m}R_{i}^{2}-m{\left(m+1\right)}^{2}\right)}{4\sum_{i=1}^{m}\sum_{j=1}^{N}r_{ji}^{2}-Nm{\left(m+1\right)}^{2}}.$$

Test statistics under null hypothesis that all classifiers have the same performance follows the $\chi^{2}$ distribution with m−1 degrees of freedom. *p*-value of this test is the probability of observing by chance the same or greater $\chi_{F}^{2}$ if all classifiers have the same performance. *p*-value is $p_{\chi }=1-F\left(\chi_{F}^{2};m-1\right)$, where F(χ;df) is the cumulative $\chi^{2}$ distribution with df degrees of freedom. Since we only have 37 databases, we decide to use the 95% significance level.

If the Friedman test shows enough evidence to reject the null hypothesis, then we can conclude that not all classifiers have the same performance. To identify pairs of classifiers with significantly different performances, we applied the post hoc Nemenyi test [110]. Test statistics for comparing of classifiers *i* and *j* is $|R_{i}-R_{j}|$. To identify pairs with statistically significant differences the critical distance

(16) $$\mathrm{CD}=q_{\alpha m}\sqrt{\frac{m(m+1)}{6N}}.$$

is used. Here, $q_{\alpha m}$ is the critical value for the Nemenyi test with a significance level of 1−α and *m* degrees of freedom. The difference of classifiers performances is statistically significant with a significance level of 1−α if $|R_{i}-R_{j}|>CD$.

#### 4.2.3. Wilcoxon Signed Rank Test

To compare the performance of two classifiers on several databases we applied the Wilcoxon signed rank test [115]. For this test we used the standard Matlab function **signrank** [116].

## 5. Dimension Comparison

An evaluation of six dimensions, number of attributes (dimension of space) and five intrinsic dimensions of data, for benchmarks is presented in Table 2. It can be seen, that for each considered intrinsic dimension of data, this dimension does not grow monotonously with the number of attributes for the given set of benchmarks. The correlation matrix of all six dimensions is presented in Table 3. There are two groups of highly correlated dimensions:

- #Attr, PCA-K and PCA-BS;
- PCA-CN and SepD.

Correlations between groups are low (the maximum value is 0.154). The fractal dimension (FracD) is correlated (but is not strongly correlated) with PCA-CN and SepD.

Consider the first group of correlated dimensions. Linear regressions of PCA-K and PCA-BS on #Attr are

(17) $$\begin{array}{cc} \mathrm{PCA}\text{-}K & \approx 0.29\#\mathrm{Attr},\\ \mathrm{PCA}\text{-}\mathrm{BS} & \approx 0.027\#\mathrm{Attr}. \end{array}$$

It is necessary to emphasize that a coefficient 0.29 (0.027 for PCA-BS) was determined only for datasets considered in this study and can be different for another datasets, but multiple R squared equals 0.998 (0.855 for PCA-BS), shows that this dependence is not accidental. What is the reason for the strong correlations of these dimensions? It can be shown that these dimensions are sensitive to irrelevant or redundant attributes. The simplest example is adding highly correlated attributes. To illustrate this property of these dimensions, consider an abstract database *X* with *d* standardised attributes and a covariance matrix Σ. This covariance matrix has *d* eigenvalues $\lambda_{1}\geq \lambda_{2}\geq \ldots \geq \lambda_{d}$ and corresponding eigenvectors $v_{1},\ldots ,v_{d}$. To determine the PCA-K dimension, we must compare FVE of each principal component with the threshold 1/d. Since all attributes are standardized, the elements of the main diagonal of the matrix Σ are equal to one. This means that $\sum_{i=1}^{d}\lambda_{i}=d$ and the FVE of *i* principal component is $f_{i}=\frac{\lambda_{i}}{\sum_{j=1}^{d}\lambda_{j}}=\lambda_{i}/d$.

Consider duplication of attributes: add copies of the original attributes to the data table. This operation does not add any information to the data and, in principle, should not affect the intrinsic dimension of the data for any reasonable definition.

Denote all object for this new database by superscript (1). The new dataset is $X^{\left(1\right)}=X|X$, where symbol | denotes the concatenation of two row vectors. For any data vectors $x^{\left(1\right)}$ and $y^{\left(1\right)}$, the dot product is $\left(x^{\left(1\right)},y^{\left(1\right)}\right)=2\left(x,y\right)$.

For a new dataset $X^{\left(1\right)}$ the covariance matrix has the form

(18) $$\Sigma^{\left(1\right)}=\left[\begin{array}{cc} \Sigma & \Sigma \\ \Sigma & \Sigma \end{array}\right].$$

The first *d* eigenvectors can be represented as $v_{i}^{\left(1\right)}={\left(v_{i}^{⊺}|v_{i}^{⊺}\right)}^{⊺}$, where ⊺ means transposition of the matrix (vector). Calculate the product of $v_{i}^{\left(1\right)}$ and $\Sigma^{\left(1\right)}$:

(19) $$\Sigma^{\left(1\right)}v_{i}^{\left(1\right)}=\left[\begin{array}{cc} \Sigma & \Sigma \\ \Sigma & \Sigma \end{array}\right]\left(\begin{array}{c} v_{i}\\ v_{i} \end{array}\right)=\left(\begin{array}{c} \Sigma v_{i}+\Sigma v_{i}\\ \Sigma v_{i}+\Sigma v_{i} \end{array}\right)=\left(\begin{array}{c} \lambda_{i}v_{i}+\lambda_{i}v_{i}\\ \lambda_{i}v_{i}+\lambda_{i}v_{i} \end{array}\right)=2\lambda_{i}\left(\begin{array}{c} v_{i}\\ v_{i} \end{array}\right)=2\lambda_{i}v_{i}^{\left(1\right)}.$$

As we can see, each of the first *d* eigenvalues become twice as large ($\lambda_{i}^{\left(1\right)}=2\lambda_{i},\forall i\leq d$). This means that the FVE of the first *d* principal components have the same values

(20) $$f_{i}^{\left(1\right)}=\frac{\lambda_{i}^{\left(1\right)}}{2d}=\frac{2\lambda_{i}}{2d}=\frac{\lambda_{i}}{d}=f_{i},\forall i\leq d.$$

Since sum of the eigenvalues of the matrix $\Sigma^{\left(1\right)}$ is 2d, we can conclude that $\lambda_{i}^{\left(1\right)}=0,\forall i>d$. We can repeat the described procedure for copying attributes several times and determine the values $\lambda_{i}^{\left(m\right)}=m\lambda_{i},f_{i}^{\left(m\right)}=f_{i}\forall i\leq d$ and $\lambda_{i}^{\left(m\right)}=0,\forall i>d$, where *m* is the number of copies of attributes added. For the database $X^{\left(m\right)}$, the informativeness threshold of principal components is $\frac{1}{(m+1)d}$. Obviously, for any nonzero eigenvalue $\lambda_{i}>0$, there exists *m* such that $\lambda_{i}>\frac{1}{(m+1)d}$. This means that trivial operation of adding copies of attributes can increase informativeness of principal components and the number of informative main components or PCA-K dimension.

To evaluate the effect of the attribute copying procedure on the broken stick dimension, the following two propositions are needed:

**Proposition** **1.** *If d=2k, then $b_{k+s}^{\left(1\right)}>b_{k+s},s=1,\ldots ,k$ and $b_{k-s}^{\left(1\right)}<b_{k-s},s=0,\ldots ,k-1$.*

**Proposition** **2.** *If d=2k+1, then $b_{k+s}^{\left(1\right)}>b_{k+s},s=2,\ldots ,k+1$ and $b_{k-s}^{\left(1\right)}<b_{k-s},s=-1,\ldots ,k-1$.*

Proofs of these propositions are presented in Appendix A.

The simulation results of process of the attribute copying for ‘Musk 1’ and ‘Gisette’ databases are presented in Table 4.

Now we are ready to evaluate the effect of duplication of attributes on the dimensions under consideration, keeping in mind that nothing should change for reasonable definitions of data dimension.

- The dimension of the vector space of the dataset $X^{\left(m\right)}$ is (m+1)d (see Table 4).
- For the dimension defined by the Kaiser rule, PCA-K, the threshold of informativeness is 1/(m+1)d. This means that for all principal components with nonzero eigenvalues, we can take large enough *m* to ensure that these principal components are “informative” (see Table 4). The significance threshold decreases linearly with increasing *m*.
- For the dimension defined by the broken stick rule, PCA-BS, we observe initially an increase in the thresholds for the last half of the original principal components, but then the thresholds $b_{i}^{\left(m\right)}$ decrease with an increase in *m* for all i≤d. This means that for all principal components with nonzero eigenvalues, we can take large enough *m* to ensure that these principal components are “informative” (see Table 4). The thresholds of significance decrease non-linearly with increasing *m*. This slower than linear thresholds decreasing shows that PCA-BS is less sensitivity to irrelevant attributes than #Attr or PCA-K.
- For the PCA-CN dimension defined by condition number, nothing changes in the described procedure since simultaneous multiplying of all eigenvalues by a nonzero constant does not change the fraction of eigenvalues in the condition (7).
- Adding irrelevant attributes does not change anything for separability dimension, SepD, since the dot product of any two data points in the extended database is the dot products of the corresponding vectors in the original data set multiplied by m+1. This means that described extension of dataset change nothing in the separability inequality (8).
- There are no changes for the fractal dimension FracD, since the described extension of dataset does not change the relative location of data points in space. This means that values N(r) will be the same for original and extended datasets.

The second group of correlated dimensions includes PCA-CN, SepD, and FracD. The first two are highly correlated and the last one is moderately correlated with the first two. Linear regressions of these dimensions are

(21) $$\begin{array}{cc} \mathrm{SepD} & \approx 1.17\mathrm{PCA}\text{-}\mathrm{CN},\\ \mathrm{FracD} & \approx 0.052\mathrm{PCA}\text{-}\mathrm{CN}. \end{array}$$

High correlation of these three dimensions requires additional investigations.

## 6. Results of *l_p_* Functionals Comparison

The results of a direct comparison of the algorithms are presented in Table 5 for 11NN, Table A1 for 3NN, Table A2 for 5NN, and Table A3 for 7NN. Table 5 shows that ‘The best’ indicator is not reliable and cannot be considered as a good tool for performance comparison [107]. For example, for TNNSC with empty preprocessing, $l_{0.1}$ is the best for 11 databases and this is the maximal value, but $l_{0.5},l_{1}$ and $l_{2}$ are essentially better if we consider indicator ‘Insignificantly different from the best’: 26 databases for $l_{0.1}$ and 31 databases for $l_{0.5},l_{1}$ and $l_{2}$. This fact confirms that the indicator ‘Insignificantly different from the best’ is more reliable. Analysis of Table 5 shows that on average $l_{0.5},l_{1},l_{2}$ and $l_{4}$ are the best and $l_{0.01}$ and $l_{\infty }$ are the worst. Qualitatively the same results are contained in Table A1 for 3NN, Table A2 for 5NN, and Table A3 for 7NN

The results of the Friedman and post hoc Nemenyi tests are presented in Table 6, Table 7, Table 8 and Table 9. We applied these tests for three different preprocessings and three classification quality indicators. In total, we tested nine sets for eight algorithms and 37 databases. Tests was performed for kNN with k=3,5,7,11. The post hoc Nemenyi test was used to define algorithms with performance that do not significantly differ from the best algorithm. It can be seen that $l_{1}$ is the best for 50% tests (18 of 36 sets), $l_{0.5}$ is the best for 42% of tests (15 of 36 sets), and $l_{2}$ is the best for 8% of tests (3 of 36 sets). On the other hand, performances of $l_{0.5},l_{1}$ and $l_{2}$ are insignificantly different from the best for all nine sets and all four kNN.

We compared eight different $l_{p}$ functionals on 37 databases. The authors of [14] have hypothesised that: (i) kNN based on $l_{1}$ is better than based on $l_{2}$ and (ii) that the “fractional” metrics can further improve performance. We tested the differences between 11NN classifiers based on $l_{0.5},l_{1}$ and $l_{2}$ by direct usage of Wilcoxon test. This comparison does not take into account the multiple testing. The results of comparisons are presented in Table 10 and Table 11. The top table shows that in all cases kNN based on $l_{0.5}$ and $l_{1}$ have insignificantly different performances and for the most cases kNN based on $l_{2}$ is slightly worse than the previous two. The bottom table shows, that kNN based on $l_{0.5}$ and $l_{2}$ are insensitive to type of preprocessing (the performances of both methods are not significantly different for different preprocessing). In contrast to these two methods, kNN based on $l_{1}$ shows significantly better performance for min-max normalization preprocessing in comparison with two other preprocessings (*p*-values for both tests are less than 1%).

## 7. Discussion

In this paper, we tested the rather popular hypothesis that using the $l_{p}$ norms with p<2 (preferably p=1) or even the $l_{p}$ quasinorm with 0<p<1 helps to overcome the curse of dimensionality.

Traditionally, the first choice of test datasets for analysing the curse or blessing of dimensionality is to use samples from some simple distributions: uniform distributions on the balls, cubes, other convex compacts, or normal distributions (see, for example, [4,11,12,13,14,15,18], etc.). Further, generalisations are used such as the product of distributions in a cube (instead of uniform distributions) or log-concave distributions (instead of normal distributions) [28,117,118]. For such distributions was proven properties of data concentration in thin layer [117], and further in waists of such layers [22]. We used data sampled from the uniform distribution on the unit cube to analyse the distribution of $l_{p}$ distances in high dimensions for various *p*. To assess the impact of dimension on classification, we used collection of 25 datasets from different sources (Table 2). The number of attributes in these databases varies from 4 to 5000.

For real-life datasets, the distributions are not just unknown—there is doubt that the data are sampled from a more or less regular distribution. Moreover, we cannot always be sure that the concepts of probability distribution and statistical sampling are applicable. If we want to test any hypothesis about the curse or blessing of dimensionality and methods of working with high-dimensional data, then the first problem we face is: what is data dimensionality? Beyond hypotheses about regular distribution, we cannot blindly assume that the data dimensionality is the same as the number of attributes. Therefore, the first task was to evaluate the intrinsic dimension of all the data sets selected for testing.

Five dimensionalities of data were considered and compared:

- PCA with Kaiser rule for determining the number of principal components to retain (PCA-K);
- PCA with the broken stick rule for determining the number of principal components to retain (PCA-BS);
- PCA with the condition number criterion for determining the number of principal components to retain (PCA-CN);
- The Fisher separability dimension (SepD);
- The fractal dimension (FracD).

We demonstrated that both the Kaiser rule (PCA-K) and the broken stick rule (PCA-BS) are very sensitive to the addition of attribute duplicates. It can be easily shown that these dimensions are also very sensitive to adding of highly correlated attributes. In particular, for these rules, the number of informative principal components depend on the ‘tail’ of the minor components.

The condition number criterion (PCA-CN) gives much stabler results. The dimensionality estimates based on the fundamental topological and geometric properties of the data set (the Fisher separability dimension, SepD, and the fractal dimension, FracD) are less sensitive to adding highly correlated attributes and insensitive to duplicate attributes.

Dimensions PCA-K and PCA-BS are strongly correlated (r>0.9) for the selected set of benchmarks. Their correlations with the number of attributes are also very strong (Table 3). The correlations of these dimensions with three other dimensions (PCA-CN, SepD, and FracD) are essentially weaker. Dimensions PCA-CN and SepD are also strongly correlated (r>0.9), and their correlations with FracD are moderate (see Table 3).

The results of testing have convinced us that the PCA-CN and SepD estimates of the intrinsic dimensionality of the data are more suitable for practical use than the PCA-K and PCA-BS estimates. The FracD estimate is also suitable. A detailed comparison with many other estimates is beyond the scope of this paper.

The choice of criteria is very important for identifying the advantages of using non-Euclidean norms and quasi-norms $l_{p}$ (2>p>0). RC (2) and CV (3) of high dimensional data are widely used for this purposes. In some examples (see [12,14]) it was demonstrated that for $l_{p}$ norms or quasinorms, the RC decreases with increasing dimension. It was also shown [14] that RC for $l_{p}$ functionals with lower *p* are greater than for $l_{p}$ functionals with greater *p* (see Figure 3).

Our tests for data sets sampled from a regular distribution (uniform distribution in a cube) confirm this phenomenon. However Figure 3 shows that decreasing of *p* cannot compensate (improve) the curse of dimensionality: the RC for high dimensional data and small *p* will be less than for usual Euclidean distance in some lower dimensional space. The behavior of a CV with a change in dimension is similar to that of RC. In our experiments, the inequalities $RC_{p}<RC_{q},\forall p>q$ and $CV_{p}<CV_{q},\forall p>q$ were almost always satisfied. We found that the differences in RC and CV for different *p* decay with dimension tends to infinity.

Authors of [14] stated that “fractional distance metrics can significantly improve the effectiveness of standard clustering algorithms”. In contrast, our tests on the collection of the benchmark datasets showed that there is no direct relationship between the distance concentration indicators (e.g., RC or CV) and the quality of classifiers: kNN based on $l_{0.01}$ has one of the worst classification performance but the greatest RC and CV. Comparison of the classification quality of 3NN, 5NN, 7NN and 11NN classifiers for different $l_{p}$ functionals and for different databases shows that the greater RC does not mean the higher quality.

The authors of [14] found that $l_{1}$ “is consistently more preferable than the Euclidean distance metric for high dimensional data mining applications”. Our study partially confirmed the first finding: kNN with $l_{1}$ distance often shows better performance compared to $l_{0.01},l_{0.1},l_{0.5},l_{2},l_{4},l_{10},l_{\infty }$ but this difference is not always statistically significant.

Finally, the performance of kNN classifiers based on $l_{0.5},l_{1}$ and $l_{2}$ functionals is statistically indistinguishable for k=3,5,7,11.

A detailed pairwise comparison of the $l_{0.5},l_{1}$, and $l_{2}$ functions shows that the performance of a $l_{1}$ based kNN is more sensitive to the preprocessing used than a $l_{0.5}$ and $l_{2}$ based kNN. There is no unique and unconditional leader among the $l_{p}$ functionals for classification problems. We can conclude that the $l_{p}$ based kNN classifiers with very small p<0.1 and very big p>4 are almost always worse than with intermediate *p*, 0.1≤p≤4. Our massive test shows that for all preprocessing used and all considered classifier quality indicators, the performance of kNN classifiers based on $l_{p}$ for $l_{0.5}$, $l_{1}$ and $l_{2}$ does not differ statistically significantly.

In regards to the estimation of dimensions, the question is: can the number of $l_{2}$ based major principal components be considered as a reasonable estimate of the “real” data dimension or it is necessary to use $l_{1}$ based PCA? Recently developed PQSQ PCA [49] gives the possibility to create PCA with various subquadratic functionals, including $l_{p}$ for 0<p≤2. The question about performance of clustering algorithms with different $l_{p}$ functionals remains still open. This problem seems less clearly posed than for supervised classification, since there are no unconditional criteria for “correct clustering” (or too many criteria that contradict each other), as is expected for unsupervised learning.

## 8. Conclusions

Thus, after detailed discussion, we have to repeat the title “Fractional norms and quasinorms do not help to overcome the curse of dimensionality“. The ‘champion’ norms for the kNN classification are not far from the classical $l_{1}$ and $l_{2}$ norms. We did not find any evidence that it is more efficient to use in classification $l_{p}$ norms with p<1 or p>2.

What do all these results mean for the practice of data mining? The fist answer is: we have to trust in classical norms more. If there are no good classifiers with the classical norms $l_{1}$ and $l_{2}$, then class separability is likely to be unsatisfactory in other norms. Feature selection and various dimensionality reduction techniques are potentially much more powerful in improving classification than playing with norms.

Of course, without any hypothesis about data distribution such an advice cannot be transformed into a theorem, but here we would like to formulate a hypothesis that for sufficiently regular distributions in classes, the performance of kNN classifiers in high dimensions is asymptotically the same for different $l_{p}$ norms.

What can we say about other data mining problems? We cannot be sure a priori that the change of norm will not help. Nevertheless, the geometric measure concentration theorems give us a hint that for sufficiently high dimensionality of data the difference between the methods that use different norms will vanish.

Of course, this advice also has limitations. There are some obvious differences between norms. For example, partial derivatives of $l_{1}$ and $l_{2}$ norms $\partial ∥x∥/\partial x_{i}$ differ significantly at zeros of coordinate $x_{i}$. This difference was utilised, for example, in lasso methods to obtain sparse regression [119]. These properties are not specific for high dimension.

## Figures and Tables

**Table 1:** Comparison of RC for $l_{1}$ and $l_{2}$ for different dimension of space (Dim) and different number of points.

Dim	$P({\mathrm{RC}}_{2}<{\mathrm{RC}}_{1})$ for # of Points			
	10 [14]	10	20	100
1	0	0	0	0
2	0.850	0.850	0.960	1.00
3	0.887	0.930	0.996	1.00
4	0.913	0.973	0.996	1.00
10	0.956	0.994	1.00	1.00
15	0.961	1.000	1.00	1.00
20	0.971	0.999	1.00	1.00
100	0.982	1.000	1.00	1.00

**Table 2:** Databases selected for analysis.

Name	Source	#Attr.	Cases	PCA-K	PCA-BS	PCA-CN	SepD	FracD
Blood	[74]	4	748	2	2	3	2.4	1.6
Banknote authentication	[75]	4	1372	2	2	3	2.6	1.9
Cryotherapy	[76,77,78]	6	90	3	0	6	4.1	2.5
Vertebral Column	[79]	6	310	2	1	5	4.4	2.3
Immunotherapy	[76,77,80]	7	90	3	0	7	5.1	3.2
HTRU2	[81,82,83]	8	17,898	2	2	4	3.06	2.4
ILPD (Indian LiverPatient Dataset)	[84]	10	579	4	0	7	4.3	2.1
Planning Relax	[85]	10	182	4	0	6	6.09	3.6
MAGIC Gamma Telescope	[86]	10	19,020	3	1	6	4.6	2.9
EEG Eye State	[87]	14	14,980	4	4	5	2.1	1.2
Climate Model SimulationCrashes	[88]	18	540	10	0	18	16.8	21.7
Diabetic Retinopathy Debrecen	[89,90]	19	1151	5	3	8	4.3	2.3
SPECT Heart	[91]	22	267	7	3	12	4.9	11.5
Breast Cancer	[92]	30	569	6	3	5	4.3	3.5
Ionosphere	[93]	34	351	8	4	9	3.9	3.5
QSAR biodegradation	[94,95]	41	1055	11	6	15	5.4	3.1
SPECTF Heart	[91]	44	267	10	3	6	5.6	7
MiniBooNE particleidentification	[96]	50	130,064	4	1	1	0.5	2.7
First-order theorem proving(6 tasks)	[97,98]	51	6118	13	7	9	3.4	2.04
Connectionist Bench (Sonar)	[99]	60	208	13	6	11	6.1	5.5
Quality Assessment ofDigital Colposcopies (7 tasks)	[100,101]	62	287	11	6	9	5.6	4.7
LFW	[102]	128	13,233	51	55	57	13.8	19.3
Musk 1	[103]	166	476	23	9	7	4.1	4.4
Musk 2	[103]	166	6598	25	13	6	4.1	7.8
Madelon	[104,105]	500	2600	224	0	362	436.3	13.5
Gisette	[104,106]	5000	7000	1465	133	25	10.2	2.04

**Table 3:** Correlation matrix for six dimensionality: two groups of highly correlated dimensions are highlighted by the background colours.

Dimension	#Attr	PCA-K	PCA-BS	PCA-CN	SepD	FracD
#Attr	1.000	0.998	0.923	0.098	0.065	−0.081
PCA-K	0.998	1.000	0.917	0.154	0.119	−0.057
PCA-BS	0.923	0.917	1.000	0.018	−0.058	0.075
PCA-CN	0.098	0.154	0.018	1.000	0.992	0.405
SepD	0.065	0.119	−0.058	0.992	1.000	0.343
FracD	−0.081	−0.057	0.075	0.405	0.343	1.000

**Table 4:** Attribute duplication process for ‘Musk 1’ and ‘Gisette’ databases.

	Musk			Gizette		
m	#Attr	PCA-K	PCA-BS	#Attr	PCA-K	PCA-BS
0	166	23	9	4971	1456	131
1	332	34	16	9942	2320	1565
2	498	40	23	14,913	2721	1976
3	664	45	28	19,884	2959	2217
4	830	49	32	24,855	3122	2389
5	996	53	33	29,826	3242	2523
10	1826	63	39	54,681	3594	2909
50	8466	94	62	253,521	4328	3641
100	16,766	109	73	502,071	4567	3926
500	83,166	139	102	2,490,471	4847	4491
1000	166,166	150	115	4,975,971	4863	4664
5000	830,166	163	141	24,859,971	4865	4852
10,000	1,660,166	166	151	49,714,971	4866	4863

**Table 5:** Frequency comparison for TNNSC, accuracy and sensitivity plus specificity, 11NN.

Indicator\p for $l_{p}$ Functional	0.01	0.1	0.5	1	2	4	10	*∞*
**TNNSC**								
Empty preprocessing								
The best	2	11	5	10	7	1	1	1
Insignificantly different from the best	17	26	31	31	31	30	23	22
The worst	19	0	1	0	1	3	4	8
Insignificantly different from the worst	34	23	17	19	21	21	25	29
Standardisation								
The best	0	5	10	11	6	2	1	1
Insignificantly different from the best	19	26	33	32	31	30	25	24
The worst	18	2	0	0	1	2	4	10
Insignificantly different from the worst	35	24	20	19	20	21	25	28
Min-max normalization								
The best	1	5	10	13	4	6	1	3
Insignificantly different from the best	19	26	32	31	30	29	26	26
The worst	23	4	2	2	3	3	4	7
Insignificantly different from the worst	36	24	22	21	22	22	26	26
**Accuracy**								
Empty preprocessing								
The best	3	9	9	15	6	5	1	2
Insignificantly different from the best	29	31	34	35	35	35	33	30
The worst	13	3	1	2	4	4	9	14
Insignificantly different from the worst	35	32	28	28	29	29	30	31
Standardisation								
The best	2	5	12	18	7	3	1	1
Insignificantly different from the best	30	31	34	34	33	31	32	30
The worst	13	4	0	0	2	6	7	13
Insignificantly different from the worst	35	32	29	29	30	31	33	33
**Accuracy**								
Min-max normalization								
The best	2	7	15	8	8	3	3	6
Insignificantly different from the best	30	31	34	33	33	32	31	32
The worst	18	6	3	4	5	9	8	8
Insignificantly different from the worst	36	33	31	31	31	32	33	32
**Sensitivity plus specificity**								
Empty preprocessing								
The best	4	8	7	12	7	5	1	1
The worst	14	2	1	1	3	5	8	12
Standardisation								
The best	4	7	8	15	7	2	1	0
The worst	13	3	0	0	2	5	4	15
Min-max normalization								
The best	5	8	13	6	9	3	4	5
The worst	15	4	2	3	3	7	8	13

**Table 6:** Results of the Friedman test and post hoc Nemenyi test, 11NN.

Preprocessing	Quality Indicator	Friedman’s *p*-Value	The Best $l_{p}$		Set of Insignificantly Different from the Best							
			*p*	$R_{i}$	0.01	0.1	0.5	1	2	4	10	*∞*
Empty	TNNSC	<0.0001	1	6.2639		X	X	X	X	X		
	Accuracy	<0.0001	1	6.2639		X	X	X	X			
	Se+Sp	<0.0001	0.5	6.0556		X	X	X	X			
Standardisation	TNNSC	<0.0001	1	6.6944			X	X	X			
	Accuracy	<0.0001	1	6.8056			X	X	X			
	Se+Sp	<0.0001	1	6.4722		X	X	X	X			
Min-max normalization	TNNSC	<0.0001	1	6.4722			X	X	X	X		
	Accuracy	<0.0001	0.5	6.0000		X	X	X	X			
	Se+Sp	<0.0001	0.5	6.0000		X	X	X	X			

**Table 7:** Results of the Friedman test and post hoc Nemenyi test, 3NN.

Preprocessing	Quality Indicator	Friedman’s *p*-Value	The Best $l_{p}$		Set of Insignificantly Different from the Best							
			*p*	$R_{i}$	0.01	0.1	0.5	1	2	4	10	*∞*
Empty	TNNSC	<0.0001	0.5	6.0294		X	X	X	X	X		
	Accuracy	<0.0001	0.5	5.9265		X	X	X	X			
	Se+Sp	<0.0001	0.5	5.7353		X	X	X	X	X		
Standardisation	TNNSC	<0.0001	1	6.2941			X	X	X	X		
	Accuracy	<0.0001	0.5	6.3235			X	X	X	X		
	Se+Sp	<0.0001	0.5	6.1324		X	X	X	X	X		
Min-max normalization	TNNSC	<0.0001	2	6.0588			X	X	X	X		
	Accuracy	<0.0001	1	6.0000		X	X	X	X	X		
	Se+Sp	<0.0001	1	6.0147		X	X	X	X	X		

**Table 8:** Results of the Friedman test and post hoc Nemenyi test, 5NN.

Preprocessing	Quality Indicator	Friedman’s *p*-Value	The Best $l_{p}$		Set of Insignificantly Different from the Best							
			*p*	$R_{i}$	0.01	0.1	0.5	1	2	4	10	*∞*
Empty	TNNSC	<0.0001	0.5	5.9118		X	X	X	X	X		
	Accuracy	<0.0001	0.5	5.8971		X	X	X	X	X		
	Se+Sp	<0.0001	0.5	5.9853		X	X	X	X			
Standardisation	TNNSC	<0.0001	1	6.1471			X	X	X	X		
	Accuracy	<0.0001	0.5	6.1618		X	X	X	X			
	Se+Sp	<0.0001	1	6.1765		X	X	X	X			
Min-max normalization	TNNSC	<0.0001	2	6.0588			X	X	X	X		
	Accuracy	<0.0001	1	6.0000		X	X	X	X	X		
	Se+Sp	<0.0001	1	6.0147		X	X	X	X	X		

**Table 9:** Results of the Friedman test and post hoc Nemenyi test, 7NN.

Preprocessing	Quality Indicator	Friedman’s *p*-Value	The Best $l_{p}$		Set of Insignificantly Different from the Best							
			*p*	$R_{i}$	0.01	0.1	0.5	1	2	4	10	*∞*
Empty	TNNSC	<0.0001	1	6.1618		X	X	X	X	X		
	Accuracy	<0.0001	0.5	5.8971		X	X	X	X			
	Se+Sp	<0.0001	1	5.8971		X	X	X	X			
Standardisation	TNNSC	<0.0001	1	6.5147			X	X	X	X		
	Accuracy	<0.0001	0.5	6.3971			X	X	X			
	Se+Sp	<0.0001	0.5	6.1176		X	X	X	X			
Min-max normalization	TNNSC	<0.0001	2	6.0588			X	X	X	X		
	Accuracy	<0.0001	1	6.0000		X	X	X	X	X		
	Se+Sp	<0.0001	1	6.0147		X	X	X	X	X		

**Table 10:** *p*-values of Wilcoxon test for different $l_{p}$ functions: Se+Sp stands for sensitivity plus specificity.

Preprocessing	Quality Indicator	*p*-Value for $l_{p}$ and $l_{q}$		
		0.5 & 1	0.5 & 2	1 & 2
Empty	TNNSC	0.6348	0.3418	0.0469
	Accuracy	0.9181	0.0657	0.0064
	Se+Sp	0.8517	0.0306	0.0022
Standardised	TNNSC	0.3098	0.1275	0.0014
	Accuracy	0.6680	0.0202	0.0017
	Se+Sp	0.8793	0.0064	0.0011
Min-max normalization	TNNSC	0.7364	0.0350	0.0056
	Accuracy	0.1525	0.0218	0.2002
	Se+Sp	0.1169	0.0129	0.3042

**Table 11:** *p*-values of Wilcoxon test for different type of preprocessing (bottom): E for empty preprocessing, S for standardisation, and M for min-max normalization preprocessing, and Se+Sp stands for sensitivity plus specificity.

Quality Indicator	*p* of $l_{p}$ Function	*p*-Value for Pair of Preprocessings		
		E & S	E & M	S & M
TNNSC	0.5	0.5732	0.8382	0.6151
	1	0.9199	0.5283	0.1792
	2	0.9039	0.3832	0.1418
Accuracy	0.5	0.8446	0.5128	0.3217
	1	0.8788	0.0126	0.0091
	2	0.5327	0.3127	0.3436
Se+Sp	0.5	0.6165	0.2628	0.0644
	1	0.5862	0.0054	0.0067
	2	0.6292	0.3341	0.4780

## Abbreviations

The following abbreviations are used in this manuscript:

kNN	k nearest neighbours
RC	relative contrast
CV	coefficient of variation
#Attr	the number of attributes or Hamel dimension
PCA	principle component analysis
PCA-K	dimension of space according to Kaiser rule for PCA
PCA-BS	dimension of space according to broken stick rule for PCA
FVE	fraction of variance explained
PCA-CN	dimension of space defined by condition number for PCA
SepD	dimension of space defined according to separability theorem
FracD	intrinsic dimension defined as fractal dimension
TNNSC	total number of neighbours of the same class in nearest neighbours of all points
Se	sensitivity or fraction of correctly recognised cases of positive class
Sp	specificity or fraction of correctly recognised cases of negative class

## Appendix A. Proof of Propositions

**Proposition** **1.**
*If d=2k, then $b_{k+s}^{\left(1\right)}>b_{k+s},s=1,\ldots ,k$ and $b_{k-s}^{\left(1\right)}<b_{k-s},s=0,\ldots ,k-1$.*

**Proof.** From equation (6) we can write:

(A1) $$\begin{array}{cc} b_{k+s}^{\left(1\right)} & =\frac{1}{4k}\sum_{j=k+s}^{4k}\frac{1}{j}=\frac{1}{4k}\left(\sum_{j=k+s}^{2k}\frac{1}{j}+\sum_{j=k+1}^{2k}\frac{1}{2j}+\sum_{j=k+1}^{2k}\frac{1}{2j-1}\right)\\ \; & >\frac{1}{4k}\left(\sum_{j=k+s}^{2k}\frac{1}{j}+\sum_{j=k+1}^{2k}\frac{1}{2j}+\sum_{j=k+1}^{2k}\frac{1}{2j}\right)=\frac{1}{4k}\left(\sum_{j=k+s}^{2k}\frac{1}{j}+\frac{1}{2}\sum_{j=k+1}^{2k}\frac{1}{j}+\frac{1}{2}\sum_{j=k+1}^{2k}\frac{1}{j}\right)\\ \; & =\frac{1}{2}\left(\frac{1}{2k}\sum_{j=k+s}^{2k}\frac{1}{j}+\frac{1}{2k}\sum_{j=k+1}^{2k}\frac{1}{j}\right)=\frac{1}{2}\left(b_{k+s}+b_{k+1}\right)\geq \frac{1}{2}\left(b_{k+s}+b_{k+s}\right)=b_{k+s} \end{array}$$

and

(A2) $$\begin{array}{cc} b_{k-s}^{\left(1\right)} & =\frac{1}{4k}\sum_{j=k-s}^{4k}\frac{1}{j}=\frac{1}{4k}\left(\sum_{j=k-s}^{2k}\frac{1}{j}+\sum_{j=k+1}^{2k}\frac{1}{2j}+\sum_{j=k+1}^{2k}\frac{1}{2j-1}\right)\\ \; & <\frac{1}{4k}\left(\sum_{j=k-s}^{2k}\frac{1}{j}+\sum_{j=k+1}^{2k}\frac{1}{2j}+\sum_{j=k+1}^{2k}\frac{1}{2j-2}\right)=\frac{1}{4k}\left(\sum_{j=k-s}^{2k}\frac{1}{j}+\sum_{j=k+1}^{2k}\frac{1}{2j}+\sum_{j=k+1}^{2k}\frac{1}{2j}-\frac{1}{4k}\right)\\ \; & <\frac{1}{2}\left(\frac{1}{2k}\sum_{j=k-s}^{2k}\frac{1}{j}+\frac{1}{2k}\sum_{j=k+1}^{2k}\frac{1}{j}\right)=\frac{1}{2}\left(b_{k-s}+b_{k+1}\right)\leq \frac{1}{2}\left(b_{k-s}+b_{k-s}\right)=b_{k-s}. \end{array}$$

□

**Proposition** **2.**
*If d=2k+1, then $b_{k+s}^{\left(1\right)}>b_{k+s},s=2,\ldots ,k+1$ and $b_{k-s}^{\left(1\right)}<b_{k-s},s=-1,\ldots ,k-1$.*

**Proof.** From equation (6) we can write:

(A3) $$\begin{array}{cc} b_{k+s}^{\left(1\right)} & =\frac{1}{4k}\sum_{j=k+s}^{4k+2}\frac{1}{j}=\frac{1}{4k}\left(\sum_{j=k+s}^{2k+1}\frac{1}{j}+\sum_{j=k+1}^{2k+1}\frac{1}{2j}+\sum_{j=k+2}^{2k+1}\frac{1}{2j-1}\right)\\ \; & >\frac{1}{4k}\left(\sum_{j=k+s}^{2k+1}\frac{1}{j}+\sum_{j=k+1}^{2k+1}\frac{1}{2j}+\sum_{j=k+2}^{2k+1}\frac{1}{2j}\right)=\frac{1}{2}b_{k+s}+\frac{1}{4}b_{k+1}+\frac{1}{4}b_{k+2}\\ \; & >\frac{1}{2}b_{k+s}+\frac{1}{4}b_{k+s}+\frac{1}{4}b_{k+s}=b_{k+s} \end{array}$$

and

(A4) $$\begin{array}{cc} b_{k-s}^{\left(1\right)} & =\frac{1}{4k}\sum_{j=k-s}^{4k+2}\frac{1}{j}=\frac{1}{4k}\left(\sum_{j=k-s}^{2k+1}\frac{1}{j}+\sum_{j=k+1}^{2k+1}\frac{1}{2j}+\sum_{j=k+2}^{2k+1}\frac{1}{2j-1}\right)\\ \; & <\frac{1}{4k}\left(\sum_{j=k-s}^{2k+1}\frac{1}{j}+\sum_{j=k+1}^{2k+1}\frac{1}{2j}+\sum_{j=k+2}^{2k+1}\frac{1}{2j-2}\right)=\frac{1}{4k}\left(\sum_{j=k-s}^{2k+1}\frac{1}{j}+\sum_{j=k+1}^{2k+1}\frac{1}{2j}+\sum_{j=k+1}^{2k}\frac{1}{2j}\right)\\ \; & <\frac{1}{4k}\left(\sum_{j=k-s}^{2k+1}\frac{1}{j}+\sum_{j=k+1}^{2k+1}\frac{1}{2j}+\sum_{j=k+1}^{2k+1}\frac{1}{2j}\right)=\frac{1}{2}b_{k-s}+\frac{1}{2}b_{k+1}\leq b_{k-s}. \end{array}$$

□

## Appendix B. Results for kNN Tests for *k* = 3, 5, 7

**Table A1:** Frequency comparison for TNNSC, accuracy and sensitivity plus specificity, 3NN.

Indicator\p for $l_{p}$ Functional	0.01	0.1	0.5	1	2	4	10	*∞*
**TNNSC**								
Empty preprocessing								
The best	3	10	11	1	7	2	0	3
Insignificantly different from the best	23	28	31	32	32	30	27	25
The worst	20	4	1	1	1	4	4	9
Insignificantly different from the worst	32	25	23	23	24	25	26	26
Standardisation								
The best	1	4	9	7	8	2	3	2
Insignificantly different from the best	23	28	32	33	32	31	29	26
The worst	17	1	0	0	0	2	4	11
Insignificantly different from the worst	34	27	26	24	24	25	25	26
Min-max normalization								
The best	2	4	5	10	8	3	1	6
Insignificantly different from the best	18	25	31	30	28	28	25	26
The worst	21	3	2	4	2	3	3	8
Insignificantly different from the worst	33	24	24	22	21	23	26	27
**Accuracy**								
Empty preprocessing								
The best	2	11	10	2	8	1	1	2
Insignificantly different from the best	26	31	33	34	34	33	33	32
The worst	17	4	1	3	1	3	8	7
Insignificantly different from the worst	34	28	28	27	26	28	29	29
**Accuracy**								
Standardisation								
The best	0	4	12	8	8	2	3	2
Insignificantly different from the best	26	30	34	33	33	33	30	30
The worst	15	2	0	0	2	3	6	8
Insignificantly different from the worst	34	29	28	27	27	28	29	29
Min-max normalization								
The best	2	4	9	13	6	4	1	6
Insignificantly different from the best	27	29	33	32	32	31	31	31
The worst	15	4	5	4	3	4	5	9
Insignificantly different from the worst	34	30	27	28	29	29	30	29
**Sensitivity plus specificity**								
Empty preprocessing								
The best	5	11	9	4	8	2	2	1
The worst	14	2	1	2	0	2	6	9
Standardisation								
The best	1	7	10	5	8	2	2	3
The worst	12	1	0	1	2	2	7	10
Min-max normalization								
The best	4	6	9	13	6	4	4	2
The worst	14	1	2	3	2	2	5	11

**Table A2:** Frequency comparison for TNNSC, accuracy and sensitivity plus specificity, 5NN.

Indicator\p for $l_{p}$ functional	0.01	0.1	0.5	1	2	4	10	*∞*
**TNNSC**								
Empty preprocessing								
The best	2	9	7	3	7	3	2	2
Insignificantly different from the best	21	28	31	32	32	28	25	22
The worst	20	2	1	1	1	3	2	10
Insignificantly different from the worst	31	24	20	22	23	24	25	26
Standardisation								
The best	0	3	10	9	9	1	2	3
Insignificantly different from the best	20	28	32	32	31	31	28	25
The worst	20	1	1	2	0	1	2	10
Insignificantly different from the worst	34	27	21	20	21	23	26	27
Min-max normalization								
The best	2	4	5	10	8	3	1	6
Insignificantly different from the best	18	25	31	30	28	28	25	26
The worst	21	3	2	4	2	3	3	8
Insignificantly different from the worst	33	24	24	22	21	23	26	27
**Accuracy**								
Empty preprocessing								
The best	2	13	7	4	10	3	5	3
Insignificantly different from the best	26	31	32	33	33	33	32	30
The worst	15	2	1	2	4	4	7	10
Insignificantly different from the worst	33	29	27	27	26	27	27	28
Standardisation								
The best	3	11	12	8	12	3	1	3
Insignificantly different from the best	27	29	33	33	32	32	32	29
The worst	16	2	0	0	0	4	6	8
Insignificantly different from the worst	34	29	29	28	28	29	30	30
Min-max normalization								
The best	2	4	9	13	6	4	1	6
Insignificantly different from the best	27	29	33	32	32	31	31	31
The worst	15	4	5	4	3	4	5	9
Insignificantly different from the worst	34	30	27	28	29	29	30	29
**Sensitivity plus specificity**								
Empty preprocessing								
The best	5	11	7	3	8	4	4	1
The worst	12	0	0	1	3	2	7	11
Standardisation								
The best	4	11	10	5	11	1	1	2
The worst	13	0	0	0	2	2	8	9
Min-max normalization								
The best	4	6	9	13	6	4	4	2
The worst	14	1	2	3	2	2	5	11

**Table A3:** Frequency comparison for TNNSC, accuracy and sensitivity plus specificity, 7NN.

Indicator\p for $l_{p}$ functional	0.01	0.1	0.5	1	2	4	10	*∞*
**TNNSC**								
Empty preprocessing								
The best	0	9	7	5	6	3	1	4
Insignificantly different from the best	20	28	31	32	28	27	23	23
The worst	22	1	1	1	1	3	2	10
Insignificantly different from the worst	32	24	19	21	22	24	25	28
Standardisation								
The best	0	5	6	12	9	2	1	1
Insignificantly different from the best	20	26	32	32	31	31	26	25
The worst	20	2	0	0	0	2	2	9
Insignificantly different from the worst	34	26	20	20	21	22	25	28
**TNNSC**								
Min-max normalization								
The best	2	4	5	10	8	3	1	6
Insignificantly different from the best	18	25	31	30	28	28	25	26
The worst	21	3	2	4	2	3	3	8
Insignificantly different from the worst	33	24	24	22	21	23	26	27
**Accuracy**								
Empty preprocessing								
The best	4	12	13	8	8	2	2	5
Insignificantly different from the best	25	31	33	34	34	34	32	30
The worst	15	2	2	3	4	8	9	9
Insignificantly different from the worst	34	29	28	27	27	30	30	29
Standardisation								
The best	2	4	13	5	10	2	0	2
Insignificantly different from the best	27	28	34	33	32	32	31	30
The worst	14	4	1	2	1	5	6	8
Insignificantly different from the worst	34	30	29	26	28	29	31	31
Min-max normalization								
The best	2	4	9	13	6	4	1	6
Insignificantly different from the best	27	29	33	32	32	31	31	31
The worst	15	4	5	4	3	4	5	9
Insignificantly different from the worst	34	30	27	28	29	29	30	29
**Sensitivity plus specificity**								
Empty preprocessing								
The best	5	8	9	8	6	3	3	3
The worst	12	1	3	2	4	5	7	11
Standardisation								
The best	3	6	11	6	7	1	0	2
The worst	11	1	0	0	1	3	4	14
Min-max normalization								
The best	4	6	9	13	6	4	4	2
The worst	14	1	2	3	2	2	5	11
